# Feasibility of articulating laparoscopic instruments in laparoscopic gastrectomy using propensity score matching

**DOI:** 10.1038/s41598-023-44305-1

**Published:** 2023-10-13

**Authors:** So Hyun Kang, Duyeong Hwang, Mira Yoo, Eunju Lee, Young Suk Park, Sang-Hoon Ahn, Yun-Suhk Suh, Hyung-Ho Kim

**Affiliations:** 1 Department of Surgery, Seoul National University Bundang Hospital, College of Medicine, Seoul National University, 300 Gumi-Dong, Bundang-gu, Seongnam-si, 13620 Gyenggi-do Korea; 2 Department of Surgery, Chung-Ang University Gwangmyeong Hospital, Gwangmyeong, Korea; 3 Department of Surgery, Seoul National University College of Medicine, Seoul, Korea

**Keywords:** Gastric cancer, Surgical oncology

## Abstract

Advancements in minimally invasive surgery has led to the development of several surgical instruments, including the ArtiSential®. This new instrument provides a greater range of motion and improved dexterity to laparoscopic procedures, making it an alternative option to traditional straight instruments, and the Da Vinci robot system. The purpose of this study is to compare the postoperative outcomes of a prospective cohort of patients who underwent laparoscopic gastrectomy using articulating instruments with those of a historical cohort of patients who underwent the same procedure using straight instruments. The study was designed as a prospective observational cohort study matched to a retrospective historical cohort using propensity score matching. The primary outcome was the rate of early complications within 90 days of surgery. Other outcomes included the operation time, estimated blood loss, time to first flatus, time to first soft fluid diet, hospital stay, and mortality. After propensity score matching, 41 patients were enrolled in both groups. The mean age was 62.4 ± 12.3 years in the conventional group and 63.5 ± 9.6 years in the artisential group (p = 0.647). Mean operative time was significantly shorter in the artisential group compared to the conventional group (136.1 min vs. 163.9 min, p = 0.032). The time to first soft fluid diet was also significantly shorter in the artisential group (2.2 days vs. 2.8 days, p = 0.030), but there was no significant difference in the time to first flatus and overall hospital stay. The incidence of early complications was lower in the artisential group, but the difference was not significant (24.4% vs 7.3%, p = 0.070). There was no mortality in either group. The use of articulating instruments for laparoscopic gastrectomy did not increase postoperative morbidity compared to straight laparoscopic instruments. The use of articulating instruments may be associated with faster bowel recovery and less early complications.

## Introduction

Advances in minimally invasive surgery has led to the era of laparoscopic gastrectomy. Now, laparoscopic gastrectomy is a standard procedure for early gastric cancer (EGC)^1^ and several studies are supporting its role for advanced gastric cancer (AGC) as well^2^. However, although surgical techniques have improved over the years, the limited degree of freedom when using laparoscopic instruments still remains a problem in performing complex minimally invasive surgery. As a solution to this problem, robotic surgery in the form of the Da Vinci surgical platform had emerged. Robotic surgery provides articulation that mimics the wrist movement of the surgeon at the console, providing stable vision and higher precision by neutralizing hand tremor. Some studies have shown the benefits of robotic gastrectomy especially in reducing postoperative pancreatitis^3,4^. However, there are other studies that give controversial results regarding these benefits^5^, and there is still the problem of high cost and lack of tactile sense. These are major issues that robotic gastrectomy still has to overcome.

In recent years, advances in surgical technology have led to the development of articulating laparoscopic instruments, improving the precision and quality of laparoscopic procedures. The use of articulating instruments in laparoscopic gastrectomy is a relatively new area, and the true benefits of these instruments are still being studied. The ArtiSential® (LIVSMED, Korea) is a series of articulating instruments designed specifically for laparoscopic surgery. These instruments have a unique hinge mechanism that provides a greater range of motion and improved dexterity, allowing the surgeon to reach difficult areas with ease. The articulation range is similar to the Da Vinci platform. In a previous study^6^, the use of the aforementioned articulating instrument in laparoscopic gastrectomy was compared with laparoscopic gastrectomy using conventional straight instruments. The artisential group had a faster time to first soft fluid diet (p = 0.01) while there was no statistical difference in overall complications. However, there are currently no prospective data regarding the use of this novel laparoscopic device. The purpose of this paper is to compare the postoperative outcomes of a prospective cohort of patients who underwent laparoscopic gastrectomy using articulating laparoscopic instruments with those of a historical cohort of patients who underwent the same procedure using only straight instruments.

## Methods

### Study design

This study was designed as a prospective observational cohort study with a comparative analysis using a retrospective historical cohort. The prospective cohort enrolled consecutive patients who underwent laparoscopic gastrectomy for gastric cancer from May 2022 to August 2022, and the retrospective cohort included patients who underwent laparoscopic gastrectomy from May 2017 to December 2017. The time of inclusion for the historical cohort was aforementioned because the ArtiSential was used in the clinical setting from the year 2018. All surgeries were done by a single surgeon in a single center who had already overcome the learning curve for laparoscopic gastrectomy by performing about 2–300 laparoscopic gastrectomies annually for 10 years. Other inclusion criteria were as follows: confirmed adenocarcinoma from the stomach, surgery with curative intent, and those who consented to the study. Patients with distant metastasis, and those who underwent open conversion were excluded from the study. This study was approved by the institutional review board of Seoul National University Bundang Hospital (B-2209-779-103) and was performed in accordance to the 1975 Helsinki declaration. Informed consent was obtained from all study participants.

### Operative procedures

Surgery for both the conventional and artisential groups was performed with the patient in lithotomy position and the operator seated between the legs facing cephalad. Trasumbilical incision was made and a Lap-Protector (Hakko, Tokyo, Japan) was inserted and connected with an EZ Access (Hakko, Tokyo, Japan) cap attached with three trocars. Additional trocars were inserted at the LUQ and RUQ when necessary. For the artisential group, the articulating graspers and needle holders were used. Straight graspers were used when needed, but the left hand mostly used the artisential grasper. The energy devices used were all straight. Sutures were all made using the articulating needle holders. All other instruments such as the dissector and vessel clips were used as straight instruments. Drain insertion was not performed routinely.

### Perioperative management and outcomes

Perioperative management was performed using the same clinical pathway and protocol for both groups. Patient controlled anesthesia was used as routine pain control, and more analgesics were provided at patient request. Patients were given soft fluid diet with the following criteria: (1) tolerable intake of water for 24 h, (2) stable vital signs, (3) no abdominal pain at rest. If a drain was placed, it was removed after soft fluid diet was tolerable with stable vital signs. Primary outcome was set as the rate of early complications within 90 days from surgery. Other outcomes included postoperative outcome such as operation time, estimated blood loss (EBL), time to first flatus, time to first soft fluid diet, hospital stay, and mortality. Operation time was defined as time starting from the first incision to time until all dressing was completed.

### Statistical analysis

Sample size was calculated using the results of a comparative study between robotic and laparoscopic gastrectomy^7^. With an estimated complication rate of 4.8% versus 1.0%, a power of 80% and alpha of 0.05, the final sample size was 394 (including 10% drop out rate) for each group. However, since this was a phase II observational study, 40 patients were decided to be enrolled. Ten patients were expected to enroll per month, thus the enrollment period was set from May 2022 to August 2022. Propensity score matching was done by using age, sex, body mass index (BMI), American Society of Anesthesiologists (ASA) score, operation type, tumor size, preoperative T stage, and preoperative N stage as covariates. The nearest method of caliper 0.25 with a 1:1 ratio was used. After matching, continuous variables were compared using the Student’s t-test or Mann–Whitney U test, and categorical variables were compared using the χ^2^ test or Fisher’s exact test. Analysis was performed by using R 3.6.1 (R Core Team, 2019), and a p-value of < 0.05 was considered statistically significant.

## Results:

### Patient enrollment and demographics after propensity score matching

A total of 80 patients were enrolled in the conventional (historical cohort) group, and 41 patients were enrolled in the artisential (prospective cohort) group. After matching, 41 patients were enrolled in both groups. The summary of the changes in the difference of covariates before and after matching is shown in Supplementary Table S1. Table 1 shows the patient demographics of the two groups after matching. The mean age of patients in the conventional group was 62.4 ± 12.3 years, while the artisential group was 63.5 ± 9.6 years (p = 0.647). Gender distribution was same with 31 patients (75.6%) being male and 10 patients (24.4%) being female in both groups. BMI of patients in the conventional group was 24.2 ± 3.1 kg/m^2^, while the artisential group was 24.6 ± 2.8 kg/m^2^ with no significant difference (p = 0.542). There was also no difference in ASA score and history of abdominal operations. In the conventional group, 31 patients (75.6%) underwent distal gastrectomy, 6 patients (14.6%) underwent total gastrectomy, 1 patient (2.4%) underwent proximal gastrectomy, and 3 patients (7.3%) underwent pylorus-preserving gastrectomy. In the artisential group, 31 patients (75.6%) also underwent distal gastrectomy, 2 patients (4.9%) underwent total gastrectomy, 4 patients (9.8%) underwent proximal gastrectomy, and 4 patients (9.8%) underwent pylorus-preserving gastrectomy (p = 0.268).

**Table 1:** Patient characteristics and demographics of the conventional group and the ArtiSential group after propensity score matching.

Group	Conventional	Artisential	p-value
	(N = 41)	(N = 41)	
Age (years)	62.4 ± 12.3	63.5 ± 9.6	0.647
Sex			
Male	31 (75.6%)	31 (75.6%)	1.000
Female	10 (24.4%)	10 (24.4%)	
Body mass index (kg/m^2^)	24.2 ± 3.1	24.6 ± 2.8	0.542
ASA score*			
1	12 (29.3%)	6 (14.6%)	0.167
2	23 (56.1%)	31 (75.6%)	
3	6 (14.6%)	4 (9.8%)	
History of abdominal operations	7 (17.1%)	9 (22.0%)	0.781
Operation type			
Distal gastrectomy	31 (75.6%)	31 (75.6%)	0.268
Total gastrectomy	6 (14.6%)	2 (4.9%)	
Proximal gastrectomy	1 (2.4%)	4 (9.8%)	
Pylorus-preserving gastrectomy	3 (7.3%)	4 (9.8%)	
Lymph node dissection			
D1	3 (7.3%)	3 (7.3%)	0.060
D1 +	14 (34.1%)	25 (61.0%)	
D2	22 (53.7%)	13 (31.7%)	
D2 +	2 (4.9%)	0 (0.0%)	
Lymphatic invasion	12 (29.3%)	13 (31.7%)	1.000
Vascular invasion	7 (17.1%)	3 (7.3%)	0.311
Perineural invasion	11 (26.8%)	7 (17.1%)	0.423
Tumor size (cm)	2.8 ± 1.6	2.8 ± 1.6	0.951
Proximal margin (cm)	5.1 ± 2.9	3.0 ± 2.0	< 0.001
Distal margin (cm)	5.7 ± 4.5	6.0 ± 4.0	0.759
T stage			
T1a	12 (29.3%)	17 (41.5%)	0.460
T1b	15 (36.6%)	11 (26.8%)	
T2	4 ( 9.8%)	6 (14.6%)	
T3	7 (17.1%)	3 (7.3%)	
T4a	3 (7.3%)	4 (9.8%)	
N stage			
N0	29 (70.7%)	28 (68.3%)	0.789
N1	5 (12.2%)	8 (19.5%)	
N2	3 (7.3%)	2 (4.9%)	
N3a	4 (9.8%)	3 (7.3%)	

There was no statistical difference in the lymph node dissection performed between the two groups, however there was a trend towards a higher proportion of patients undergoing D2 dissection in the conventional group (p = 0.060). The presence of lymphatic invasion, vascular invasion, and perineural invasion was also similar (p = 1.000, 0.311, and 0.423, respectively). There was no difference in tumor size and distal margin between the two groups (p-values = 0.951 and 0.759, respectively), but there was a significant difference in the proximal margin, with patients in the artisential group having a shorter proximal margin (5.1 ± 2.9 cm vs 3.0 ± 2.0 cm, p < 0.001). There was also no difference in T stage and N stage between the two groups (p = 0.460 and 0.789, respectively).

### Comparison of operative outcome and early complications

The operative outcomes comparing the two groups are shown in Table 2. Mean operative time was significantly shorter in the artisential group compared to the conventional group (136.1 ± 50.9 min vs. 163.9 ± 63.3 min, p = 0.032). EBL was similar between the two groups (31.5 ± 32.8 ml vs. 34.4 ± 61.9 ml, p = 0.791). The time to first soft fluid diet was significantly faster in the artisential group compared to the conventional group (2.2 ± 0.6 days vs. 2.8 ± 1.5 days, p = 0.030), but there was no difference in time to first flatus and overall hospital stay (p = 0.263 and 0.204, respectively). The average number of retrieved lymph nodes for the conventional group was 55.5 ± 16.5 and for the artisential group was 49.5 ± 14.7 (p = 0.084).

**Table 2:** Operative outcomes of the conventional group and the ArtiSential group after propensity score matching.

Group	Conventional	Artisential	p-value
	(N = 41)	(N = 41)	
Operation time (minutes)	163.9 ± 63.3	136.1 ± 50.9	0.032
Estimated blood loss (ml)	34.4 ± 61.9	31.5 ± 32.8	0.791
Hospital stay (days)	6.1 ± 2.6	7.0 ± 3.4	0.204
Time to soft fluid diet (days)	2.8 ± 1.5	2.2 ± 0.6	0.030
Time to first flatus (days)	3.3 ± 1.4	3.0 ± 0.8	0.263
Retrieved number of lymph nodes	55.5 ± 16.5	49.5 ± 14.7	0.084
Early complications	10 (24.4%)	3 (7.3%)	0.070
Pulmonary	8 (19.5%)	0 (0.0%)	
Ileus	2 (4.9%)	2 (4.9%)	
Fluid collection	1 (2.4%)	1 (2.4%)	
Early complications ≥ C-D grade IIIa	1 (2.4%)	1 (2.4%)	1.000
Mortality	0 (0.0%)	0 (0.0%)	N/A

*C–D* Clavien–Dindo.

The incidence of early complications was lower in the artisential group compared to the conventional group (3 vs. 10 patients, p-value = 0.070), but the difference was not significant. There was one patient who had fluid collection that needed to be removed through percutaneous drainage in each group, hence there was no significant difference in the incidence of early complications of Clavien-Dindo grade^8^ IIIa or higher between the two groups (p = 1.000). There was no case or readmission within 90 days from the operation, and also no case of mortality in either group.

## Discussion

This is the first report of a prospective cohort of patients where ArtiSential, a series of articulating laparoscopic instruments, was used for laparoscopic gastrectomy, and the results of this study provide insights into its use. In this comparative analysis, the use of the articulating laparoscopic instrument did not increase postoperative morbidity and mortality compared to a historical cohort of straight conventional laparoscopic instruments. These results are consistent with the results of a previous study by Lee et al.^6^. There was no statistical difference in early postoperative complications between the two groups (23% for the conventional group, and 26% for the artisential group), and also no statistical difference between early complications of Clavien-Dindo grade IIIa or higher (4.9% for the conventional group, and 8.2% for the artisential group). In this study, the complication rate of Clavien-Dindo grade IIIa or higher was 2.4% (n = 1) in both groups. Another retrospective study by Kim et al.^9^ compared the use of artisential in single-port gastrectomy to using prebent graspers. Seventeen patients who used artisential in single-port gastrectomy were compared to twenty-five who used prebent graspers. The number of postoperative complications with Clavien-Dindo grade IIIa or higher was 5.9% (n = 1) in the artisential group and 16.0% (n = 4) in the prebent grasper group. Thus, current evidence shows that the use of articulating instruments in laparoscopic gastrectomy does not increase postoperative morbidity. With laparoscopic gastrectomy as a start, reports of the use of this device in other types of surgery such as colorectal^10–12^, gynecological^13^, and pediatric surgery^14^ are increasing.

In this study, patients who underwent laparoscopy with an articulating instrument experienced faster times to soft fluid diet than those who used conventional instruments (2.8 ± 1.5 days vs 2.2 ± 0.6 days, p = 0.030). The retrospective analysis by Lee et al.^6^ also showed the same results, with the artisential group having faster time to first soft fluid diet (2.6 ± 1.3 days vs 2.3 ± 0.6 days, p = 0.015). Also, in the current study, the artisential group had shorter operation time (163.9 ± 63.3 min. vs 136.1 ± 50.9 min., p = 0.032). The difference in operation time is the possible reason for the difference in pulmonary complications between the two groups. Among the 8 patients in the conventional group with pulmonary complications, 7 were atelectasis which were managed conservatively. However, the results of these outcomes may be caused by the standardization in minimally invasive surgical skills over time—including advancements in surgical tools and techniques—and not because of the use of the articulating device itself. Also, more patients had function-preserving gastrectomy such as proximal gastrectomy and pylorus-preserving gastrectomy in the artisential group which also may have resulted in having faster operative time. The study by Kim et al.^9^ did not show any difference in postoperative hospital stay days, time to first fluid diet, and operation time. More prospective comparative studies are needed to see if the use of these devices does improve postoperative recovery for patients undergoing laparoscopic gastrectomy.

Robotic gastrectomy is a form of minimally invasive surgery that uses a surgical platform such as the Da Vinci to control the instruments during surgery. The operator sits on the console away from the patient, and remote controls the robotic arms that hold the surgical instruments. The benefits of robotic gastrectomy include improved precision and control, leading to improved surgical outcomes. The study by Uyama et al.^3^ was designed as a multi-institutional single arm prospective study included 330 patients from 15 institutions. The morbidity rate of robotic gastrectomy was compared with that of a historical control which used conventional laparoscopic gastrectomy, and robotic gastrectomy showed significant reduction in morbidity up to 2% as compared to laparoscopic gastrectomy which was 6%. The retrospective study by Omori et al.^7^ also supported the finding by using propensity score matching. Postoperative morbidity was less in the robotic gastrectomy group (1.0%) compared to laparoscopic group (4.8%, p = 0.007). Their data showed that robotic gastrectomy also had shorter operative time, less EBL, and shorter hospital stay. A large multicenter cohort study by Li et al.^15^ evaluated the data of 5402 patients in China, and results showed that there were fewer overall complications, less EBL, more retrieved lymph nodes in total, and more retrieved lymph nodes in suprapancreatic areas in patients who underwent robotic gastrectomy compared to the laparoscopic group. However, all these trials were designed as either observational or retrospective, and there is a significant amount of possible bias in comparison. The randomized controlled trial (RCT) by Ojima et al.^4^ showed conflicting results with the previously mentioned studies. In this RCT, 241 patients with clinical stages I-III gastric cancer were randomized to robotic or laparoscopic gastrectomy. Their results failed to show a statistical difference in intra-abdominal infectious complications between the robotic group (6.2%) and the laparoscopic group (8.5%). With these conflicting results and the problem in cost-effectiveness^16^, the current platform of robotic surgery may not be enough to completely replace laparoscopic surgery.

The ArtiSential instruments used here show a 7-degree of freedom in articulation similar to robotic surgery. While providing a similar degree of freedom, it also compliments the current major disadvantages of the robotic system—the lack of tactile sense. Darwich et al.^17^ showcased the benefits of this device in performing laparoscopic low anterior resection with total mesorectal excision. The possible wrist motions and methods of using of monopolar and bipolar energy for coagulation are described in detail in this report. Just as robotic gastrectomy, articulating instruments can grasp the lymph node tissues around the pancreas without pushing or manipulating the pancreas itself, which is inevitable during laparoscopic gastrectomy using straight instruments. Minimizing the manipulation and damage to pancreatic tissues is a crucial factor in reducing postoperative morbidity for laparoscopic gastrectomy^7^. The advantage of theses devices over robotic surgery is the additional tactile sense, and the operator can palpate the surrounding pancreatic tissues. In addition, there may also be less bleeding because more delicate and precise control of the tissues are possible due to its wide range of motion.

However, just as any new surgical device, there are studies examining the learning curve of this novel instrument. The study by Min et al.^18^ compared the ability of using the ArtiSential in performing challenging sutures, comparing the time it took with the robotic system. Five experienced surgeons who were new to articulating instruments, including the robotic system, performed the suture task with both instruments. The total completion time was 127 s for ArtiSential and 112 s for the robot with no significant difference (p = 0.754). In the analysis by Kim et al.^9^, the learning curve for the use of these articulating graspers in single-port gastrectomy was 5 cases. An animal study by Kim et al. compared the performance of the articulating graspers with straight laparoscopic instruments and robotic system for renal surgery using an porcine model. The results showed that the median operative times for renal pedicle clamping and ureter dissection were significantly shorter in the artisential group compared to the robotic group. However, the median operative time for bladder repair was significantly longer in the artisential group compared to the robotic and straight-shaped groups. The results from multiple studies suggest that these articulating instruments can be a viable alternative to the robotic system in some surgical procedures, but further research is needed to fully understand its benefits and limitations.

There are several limitations to this study. First, although the artisential cohort was collected prospectively, it was compared to a historical retrospective cohort. Although propensity score matching was used to try and reduce the bias in comparison, there was still a difference in the exact number for the type of operations, which possibly caused the difference in some postoperative outcomes such as operation time, bowel recovery, and tumor margin. Also, there was no analysis of cost-effectiveness in this study which is arguably the most important benefit in using the ArtiSential compared to robotic surgery. Another major drawback of this study is that all the surgical procedures were performed by a single surgeon who had already overcome the learning curve of laparoscopic gastrectomy using both the straight and articulating instruments. A larger study with several operators involved is needed to see if the results of our study can be generalized.

Overall, the use of articulating instruments in laparoscopic surgery is a promising development that has the potential to improve surgical outcomes and reduce the invasiveness of surgical procedures. This study shows that the use of these instruments in complex laparoscopic procedures such as gastrectomy is not only safe, but may also improve surgical outcomes such as postoperative morbidity and bowel recovery. Articulating laparoscopic instruments offer comparable degree of freedom and precision while addressing the issues of cost and tactile feedback associated with robotic systems. The introduction of products like ArtiSential may be a step forward in improving surgical quality, and further studies are needed to find the possible benefits of these instruments.

## Conclusion

In conclusion, the use of articulating instruments in laparoscopic gastrectomy is safe and does not increase postoperative morbidity compared to laparoscopic gastrectomy using straight instruments. Their use may be associated with faster bowel recovery and less early complications.

### Supplementary Information

Supplementary Table 1.

## Data Availability

The datasets used and/or analyzed during the current study are available from the corresponding author on reasonable request.

## References

[1] Associationjgca@koto.kpu-m.ac.jp JGC. Japanese gastric cancer treatment guidelines 2018 (5th edition). *Gastric Cancer* 1–21. 10.1007/s10120-020-01042-y (2020). 10.1007/s10120-020-01042-y PMC7790804 32060757

[2] Park DJ, Han SU, Hyung WJ (2012). Long-term outcomes after laparoscopy-assisted gastrectomy for advanced gastric cancer: A large-scale multicenter retrospective study. Surg. Endosc..

[3] Uyama I, Suda K, Nakauchi M (2019). Clinical advantages of robotic gastrectomy for clinical stage I/II gastric cancer: A multi-institutional prospective single-arm study. Gastric Cancer.

[4] Ojima T, Nakamura M, Hayata K (2021). Short-term outcomes of robotic gastrectomy vs laparoscopic gastrectomy for patients with gastric cancer. JAMA Surg..

[5] Kim H-I, Han S-U, Yang H-K (2016). Multicenter prospective comparative study of robotic versus laparoscopic gastrectomy for gastric adenocarcinoma. Ann. Surg..

[6] Lee E, Lee K, Kang SH (2021). Usefulness of articulating laparoscopic instruments during laparoscopic gastrectomy for gastric adenocarcinoma. J. Min. Invasive Surg..

[7] Omori T, Yamamoto K, Hara H (2022). Comparison of robotic gastrectomy and laparoscopic gastrectomy for gastric cancer: A propensity score-matched analysis. Surg. Endosc..

[8] Clavien PA, Barkun J, de Oliveira ML (2009). The Clavien-Dindo classification of surgical complications. Ann. Surg..

[9] Kim A, Lee CM, Park S (2021). Is it beneficial to utilize an articulating instrument in single-port laparoscopic gastrectomy?. J. Gastric Cancer.

[10] Jin HY, Ibahim AM, Bae JH (2022). Initial experience of laparoscopic complete mesocolic excision with D3 lymph node dissection for right colon cancer using Artisential®, a new laparoscopic articulating instrument. J. Minim. Access Surg..

[11] Jin HY, Lee CS, Lee YS (2021). Laparoscopic extended right hemicolectomy with D3 lymph node dissection using a new articulating instrument. Tech. Coloproctol..

[12] Darwich I, Abuassi M (2022). Single-center results of colorectal procedures performed with fully articulated laparoscopic Artisential® devices. Surg. Technol. Int..

[13] Bak S, Min SJ, Lee KH (2022). Single-site robotic, endometrial cancer staging surgery using additional laparoscopic multi-articulating instrument. Artisential. Gynecol. Robot. Surg..

[14] Parente G, Thomas E, Cravano S (2021). ArtiSential® articulated wrist-like instruments and their first application in pediatric minimally invasive surgery: Case reports and literature review of the most commonly available robot-inspired devices. Children.

[15] Li Z-Y, Zhou Y-B, Li T-Y (2023). Robotic gastrectomy versus laparoscopic gastrectomy for gastric cancer. Ann. Surg..

[16] Li J, Xi H, Guo X (2019). Surgical outcomes and learning curve analysis of robotic gastrectomy for gastric cancer: Multidimensional analysis compared with three-dimensional high-definition laparoscopic gastrectomy. Int. J. Oncol..

[17] Darwich I, Abuassi M, Aliyev R (2022). Early experience with the ARTISENTIAL® articulated instruments in laparoscopic low anterior resection with TME. Tech. Coloproctol..

[18] Min S-H, Cho Y-S, Park K (2019). Multi-DOF (degree of freedom) articulating laparoscopic instrument is an effective device in performing challenging sutures. J. Min. Invas. Surg..

