# Supplementary material for: Feasibility of articulating laparoscopic instruments in laparoscopic gastrectomy using propensity score matching
Source: Sci Rep. 2023 Oct 13;13:17384. doi: 10.1038/s41598-023-44305-1 (PMC10576043; doi:10.1038/s41598-023-44305-1)
Supplement: Supplementary file 1 — Supplementary Table 1. [file 41598_2023_44305_MOESM1_ESM.docx]

**Supplementary Table 1. Differences in covariates of the conventional and artisential group before and after propensity score matching**

| Covariates | Conventional | Artisential | p-value | Standardized Difference | Conventional | Artisential | p-value | Standardized Difference |
| --- | --- | --- | --- | --- | --- | --- | --- | --- |
|  | N=80 | N=41 |  |  | N=41 | N=41 |  |  |
| Age | 61.8 ± 12.0 | 63.5 ± 9.6 | 0.42 | 0.182 | 62.4 ± 12.3 | 63.5 ± 9.6 | 0.647 | 0.117 |
| Female gender | 31 (38.8%) | 10 (24.4%) | 0.169 | -0.33 | 10 (24.4%) | 10 (24.4%) | 1.000 | 0.000 |
| Body mass index (kg/m^2^) | 23.9 ± 3.0 | 24.6 ± 2.8 | 0.213 | 0.251 | 24.2 ± 3.1 | 24.6 ± 2.8 | 0.542 | 0.142 |
| ASA^a^ Score |  |  | 0.011 | 0.48 |  |  | 0.167 | 0.196 |
| 1 | 32 (40.0%) | 6 (14.6%) |  |  | 12 (29.3%) | 6 (14.6%) |  |  |
| 2 | 39 (48.8%) | 31 (75.6%) |  |  | 23 (56.1%) | 31 (75.6%) |  |  |
| 3 | 9 (11.2%) | 4 (9.8%) |  | -0.854 | 6 (14.6%) | 4 (9.8%) |  |  |
| Operation type |  |  | 0.030 | 0.182 |  |  | 0.268 | 0.119 |
| Distal gastrectomy | 61 (76.2%) | 31 (75.6%) |  |  | 31 (75.6%) | 31 (75.6%) |  |  |
| Total gastrectomy | 14 (17.5%) | 2 (4.9%) |  |  | 6 (14.6%) | 2 (4.9%) |  |  |
| Proximal gastrectomy | 1 (1.2%) | 4 (9.8%) |  |  | 1 (2.4%) | 4 (9.8%) |  |  |
| Pylorus-preserving gastrectomy | 4 (5.0%) | 4 (9.8%) |  |  | 3 (7.3%) | 4 (9.8%) |  |  |
| Tumor size (cm) | 4.1 ± 3.0 | 2.8 ± 1.6 | 0.001 | -0.854 | 2.8 ± 1.6 | 2.8 ± 1.6 | 0.951 | -0.014 |
| Preoperative Tstage |  |  | 0.359 | -0.372 |  |  | 0.499 | -0.148 |
| cT1 | 43 (53.7%) | 28 (68.3%) |  |  | 29 (70.7%) | 28 (68.3%) |  |  |
| cT2-4 | 37 (46.3%) | 13 (31.7%) |  |  | 12 (29.3%) | 13 (31.7%) |  |  |
| Preoperative Nstage |  |  | 0.634 | -0.307 |  |  | 1.000 | -0.054 |
| cN0 | 50 (62.5%) | 28 (68.3%) |  |  | 29 (70.7%) | 28 (68.3%) |  |  |
| cN+ | 30 (37.5%) | 13 (31.7%) |  |  | 12 (29.3%) | 13 (31.7%) |  |  |

^a^ abbreviation: ASA – American Society of Anesthesiologists

**Title:** Feasibility of articulating laparoscopic instruments in laparoscopic gastrectomy – a comparative study using propensity score matching

**Journal:** Langenbeck’s Archives of Surgery

**Authors:** So Hyun Kang, MD^a^, Duyeong Hwang, MD^a^, Mira Yoo, MD^a^, Eunju Lee, MD^b^, Young Suk Park, MD^a,c^, Sang-Hoon Ahn, MD^a,c^, Yun-Suhk Suh, MD, PhD^a,c^, Hyung-Ho Kim, MD, PhD^a,c^

**Author affiliations:**

a Department of Surgery, Seoul National University Bundang Hospital, Seongnam, Korea

b Department of Surgery, Chung-Ang University Gwangmyeong Hospital, Gwangmyeong

c Department of Surgery, Seoul National University College of Medicine, Seoul, Korea

**Correspondence to:**

Sang-Hoon Ahn, MD

Associate Professor

Department of Surgery

Seoul National University Bundang Hospital

Seoul National University, College of Medicine

300 Gumi-dong, Bundang-gu, Seongnam-si, Gyenggi-do, 13620, KOREA

Tel: +82-31-787-7106

Fax: +82-31-787-4078

Email address: viscaria@snubh.org
